# Induction of triglyceride accumulation and mitochondrial maintenance in muscle cells by lactate

**DOI:** 10.1038/srep33732

**Published:** 2016-09-20

**Authors:** Jingquan Sun, Xin Ye, Minhao Xie, Jianping Ye

**Affiliations:** 1 Sport Science College of Beijing Sport University, Beijing, China; 2 Antioxidant and Gene Regulation Laboratory, Pennington Biomedical Research Center, Louisiana State University, Louisiana, USA; 3 China Institute of Sports Medicine, Beijing, China

## Abstract

Muscle exercise induces intramuscular triglyceride (TG) accumulation and promotes mitochondrial maintenance in myotubes. However, the mechanism underlying exercise effects remains unknown. In this study, lactic acid was tested as a signaling molecule in C2C12 myotubes to understand the mechanism. Intracellular TG storage was induced in the cells by sodium lactate. The lactate activity was observed with an inhibition of the cAMP-PKA pathway as indicated by a reduction in the phosphorylation status of CREB (pCREB). Induction of pCREB signal by forskolin was blocked by pretreatment of cells with lactate. The impact of lactate on mitochondrial function was examined with a focus on the activities of two enzymes, MCAT (malonylCoA:ACP transferase) and PDH (pyruvate dehydrogenase). The enzyme activities were induced in the cells by lactate. Expression of the lactate receptor (GPR81) and lactate transporters (MCT1/4) were induced as well by lactate. The lactate activities were observed at concentrations between 4–64 mM, and were not dependent on the increase in intracellular pyruvate. Pyruvate treatment did not generate the same effects in the cells. Those results suggest that lactate may induce intramuscular TG storage and mitochondrial maintenance in myotubes through inhibition of the cAMP pathway by activation of GPR81 in a positive feedback manner.

Lactic acid is a product of glycolysis in cells and the production is enhanced under hypoxia conditions. Lactic acid exists in the sodium salt (i.e. sodium lactate) form in the body in most conditions. Lactate level is dramatically increased in the skeletal muscle by oxygen deficiency during the intensive physical exercise. Lactate concentrations may reach 10–20 mM in the circulation^1^,^2^ or even 40 mM in the skeletal muscle after the intense anaerobic exercise in humans^3^. In the classical biochemistry, lactate is used in ATP production in the tricarboxylic acid cycle in the presence of oxygen or used as a substrate in gluconeogenesis^4^. The lactate utilization is dependent on conversion into pyruvate by pyruvate dehydrogenase (PDH). Extracellular lactate is transferred into cells through its transporters, such as the proton-linked monocarboxylate transporters (MCTs). MCT1 is expressed in most cell types at a low level and MCT4 is expressed at a high level in the white skeletal muscle fibers^5^. The transporters moves lactate in and out of cells.

The signaling activity of lactate (termed lactormone) has been identified in adipocytes recently^6^, which provides a mechanism for reduction of plasma free fatty acids (FFAs) by lactate, a well-known effect of lactate. Lactate reduces FFAs by inhibition of adipocyte lipolysis. Lactate binds to the cell membrane receptor GPR81, an orphan G_i_-protein-coupled receptor in adipocytes^7^,^8^,^9^. The receptor is activated by lactate to suppress the cAMP production by down-regulation of the adenyl cyclase activity^9^. In 2008, GPR81 was identified as a receptor of lactate in adipocytes in cell culture systems^8^. In 2009, GPR81 was found to be internalized after lactate ligation^7^. The highest expression of GPR81 was found in the adipose tissue followed by skeletal muscle, liver and kidney^7^. In 2010, the GPR81 activity was confirmed in adipose tissue in a line of global GPR81 gene knockout mice^9^. In the GPR81 pathway, the receptor activation leads to inhibition of the adenylyl cyclase activity for the down-regulation of the cAMP-PKA pathway. Given the role of cAMP in the induction of lipolysis and inhibition of lipogenesis^10^, inhibition of the cAMP-PKA pathway provides a perfect model for the lactate activity in the induction of TG accumulation in adipocytes. The studies present strong evidence for the signaling activity of lactate in adipocytes. However, the signaling activity remains to be established in skeletal muscle.

Exercise training increases intramuscular TG content, which is positively associated with the muscle endurance in athletes. The intramuscular TG provides a local source of high-dense energy to support the muscle contraction^11^,^12^. Although the exercise effect on intramuscular TG has been known for a long time, the molecular mechanism is poorly understood. Based on the adipocyte studies, we propose that lactate may induce intramuscular TG accumulation by activation of the GPR81 pathway.

Mitochondrial function is required for ATP production from fatty acids, which involves β-oxidation of long chain fatty acids to generate acetyl-CoA. The mitochondrial function is dependent on many enzymes whose activities are regulated by post-translational modification from variety of small molecules. Alpha-lipoic acid (α-lipoic acid) is one of the small molecules, which is required for protein lipoylation. Lipoylation is a protein modification by adding a lipoic acid to the lysine residue of protein, which is required for the activities of pyruvate dehydrogenase (PDH) and other enzymes (such as αKDH) in mitochondria. α-lipoic acid is synthesized from acetyl-CoA by the fatty acid synthase II system (FAS II) in mitochondria^13^. MCAT (malonylCoA:ACP transferase) is a major component in the FAS II complex in mitochondria^14^,^15^. Exercise training increases the mitochondrial function, but the role of MCAT is not known in the exercise effect. In this study, lactate is tested in the regulation of MCAT activity.

In this study, we tested our lactate hypothesis in mouse C2C12 myotubes. The results suggest that lactate induces TG accumulation and MCAT expression through inhibition of the cAMP-PKA pathway by activation of GPR81. Additionally, lactate induced expression of its receptor GPR81 and lactate transporters (MCT1/4). These data suggest that lactate may serves as a signaling molecule in muscle cells by activation of GPR81 in the regulation of TG accumulation and mitochondrial function.

## Results

### Induction of TG accumulation by lactate in C2C12 myotubes

**Figure 1:**
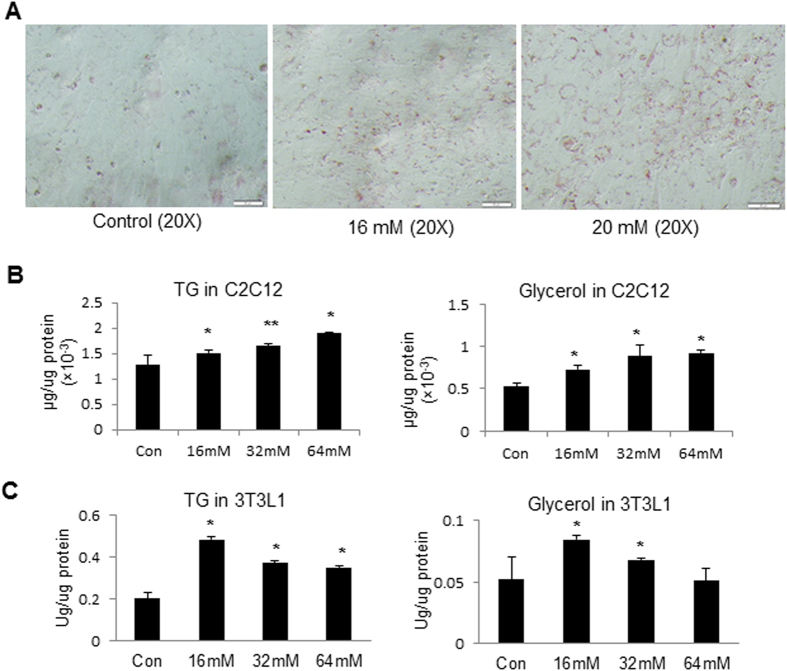
Induction of TG accumulation by lactate in C2C12 and 3T3-L1 cells. (**A**) TG in cells with oil-red O staining. C2C12 cells were treated with lactate at 16 mM and 20 mM for 4 h. Lipid accumulation was determined by oil-red O staining. (**B**) TG and glycerol quantification. C2C12 cells were treated with lactate at three concentrations for 4 h. TG and glycerol contents were determined in the whole cell lysate with the lipoprotein lipase-based colorimetric assay. (**C**) Lactate effect on adipocytes. 3T3-L1 adipocytes were treated with increasing concentrations of lactate for 4 h. TG and glycerol contents were determined with the lipoprotein lipase-based colorimetric assay. The result represents mean ± SE. *p < 0.05, **p < 0.01 compared with the control.

At the physiological concentrations, lactate induces TG accumulation in adipocytes of humans, mice, and rats^9^. To test the lactate activity in muscle cells, we treated C2C12 myotubes with lactate in cell culture and examined the intracellular TG. The TG content was significantly induced by the lactate treatment at concentrations of 16 mM and 20 mM, respectively (Fig. 1A,B). The increase was observed under microscope with oil-red O staining, and with TG quantification in the cell lysate. The increase was in a dose-dependent manner for lactate at concentrations of 16–64 mM (Fig. 1B). A similar dose-dependent effect was observed in the induction of intracellular glycerol content by lactate, a marker of TG synthesis. In the positive control, the two parameters were induced by lactate in 3T3-L1 adipocytes (Fig. 1C). The lactate activity was observed in both types of cells. However, the muscle cells and adipocytes exhibited different sensitivities to lactate. The adipocytes had a higher sensitivity as the strongest effect was observed at 16 mM of lactate. In myotubes, the strongest effect was observed at 64 mM of lactate. The data suggests that lactate induces intracellular TG accumulation in C2C12 in a dose-dependent manner at concentrations of 16–64 mM.

### Inhibition of the cAMP-PKA pathway by lactate

**Figure 2:**
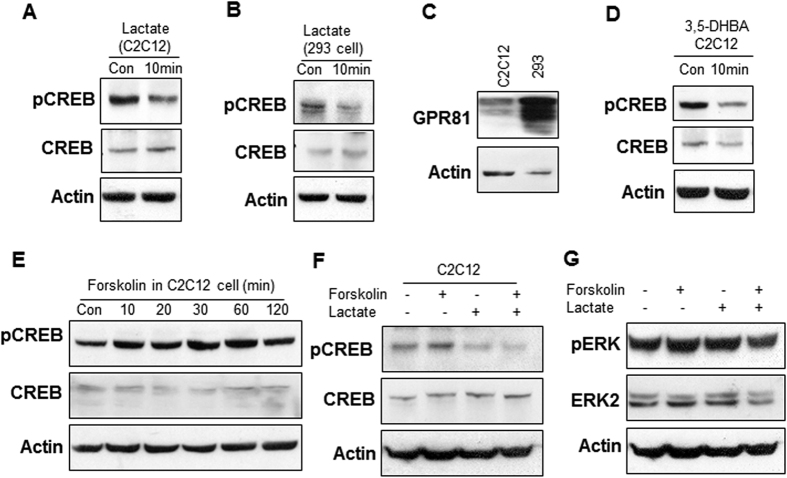
Inhibition of the cAMP-PKA pathway by lactate in C2C12. pCREB and CREB signals were determined in Western blotting. (**A**) Inhibition of the pathway in C2C12 cells by lactate (16 mM) treatment for 10 min. (**B**) Lactate activity in 293 cells. (**C**) The protein level of GPR81 in C2C12 and 293 cells. (**D**) Inhibition of pCREB signal by the GPR81 activator 3,5-DHBA. (**E**) Induction of pCREB signal in C2C12 cells by forskolin (20 μM) in a time course study. (**F**) Blockage of forskolin-induced pCREB signal with lactate (16 mM) pretreatment for 0.5 h. (**G**) Regulation of pERK signal by forskolin and lactate.

Activation of the cAMP-PKA pathway leads to lipolysis and suppression of lipogenesis^16^, which results in the reduction of intracellular TG. In the pathway, the transcription factor CREB is activated by phosphorylation on S133 to change gene expression in the lipolytic and lipogenic pathways. When the pathway is inhibited in adipocyte by lactate, the TG accumulation is increased. To test the lactate activity in muscle cells, CREB phosphorylation was examined in C2C12 myotubes. The GPR81 agonist, 3,5-DHBA and cAMP inducer forskolin were employed in the positive controls, respectively. pCREB signal was significantly reduced by lactate at 16 mM (Fig. 2A). A similar activity was observed for lactate in 293 cells (Fig. 2B). Lactate exhibited a stronger activity in 293 cells than that in C2C12 cells, which was associated with higher level of GPR81 protein in 293 cells (Fig. 2C). When GPR81 was activated with the positive control 3,5-DHBA (1 mM), a similar inhibition was observed in the pCREB signal (Fig. 2D). In contrast, pCREB signal was induced by forskolin (Fig. 2E), and the effect was blocked by lactate or 3,5-DHBA (Fig. 2F). These data suggest that lactate may activate GPR81 to inhibit the cAMP-PKA pathway in C2C12 cells. GPR81 was reported to activate the ERK signaling pathway^7^,^17^. ERK activity was examined in C2C12 cells after lactate treatment. The phosphorylation of ERK was modestly induced by lactate (Fig. 2G).

### Induction of mitochondrial MCAT activity by lactate

**Figure 3:**
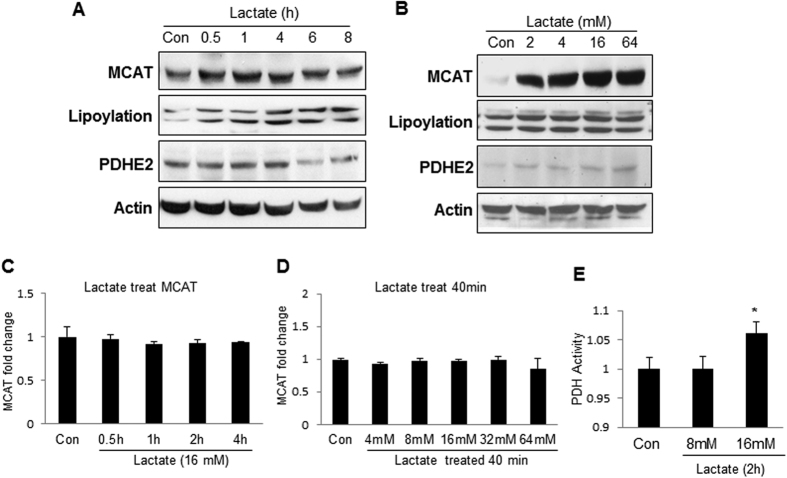
Induction of MCAT and PDH activities by lactate in C2C12 cells . (**A**) MCAT activity. MCAT protein and lipoylation of PDH were determined in C2C12 cells after lactate treatment (16 mM) in Western blot. (**B**) Induction of MCAT protein by different dosages of lactate in C2C12 cells at 4 h. (**C**) Time-dependent study of lactate effect on MCAT. mRNA was determined by qRT-PCR at different time points of lactate treatment (16 mM) in C2C12 cells. (**D**) Dose-dependent study of lactate effect on MCAT. mRNA was determined in C2C12 cells at 40 min with lactate treatment at different dosages. (**E**) Catalytic activity of PDH in C2C12 cells after lactate treatment. The test was conducted with lactate at 8 mM and 16 mM for 2 h. In the bar figure, the result represents mean ± SE (n = 3). Statistical analysis was performed with student’s test. *p < 0.05 versus control group.

MCAT is a mitochondrial protein that is required for the maintenance of mitochondrial function. MCAT promotes synthesis of α-lipoic acid in the control of lipoylation of PDH and αKDH in mitochondria^18^. MCAT was examined to investigate the potential lactate impact on mitochondrial function. The level of MCAT protein was induced by lactate (16 mM) in C2C12 cells in the time course study (Fig. 3A). The induction was observed at 30 minutes and maintained for 4 h. An increase in lipoylation was observed in PDH and αKDH in the lactate-treated cells, suggesting that the enzyme activity of MCAT was enhanced by the protein elevation. In the dose-dependent study, lactate induced MCAT protein at the concentrations of 2–64 mM in a 4 h treatment (Fig. 3B). Interestingly, MCAT mRNA was not increased by lactate in the time- and dose-dependent studies (Fig. 3C,D), suggesting a mechanism of post-translational modification in the control of MCAT protein level. As an indicator of mitochondrial function, the enzyme activity of PDH was enhanced together with the lipoylation by lactate in C2C12 cells (Fig. 3E). These data suggest that lactate may use an mRNA-independent mechanism to up-regulate MCAT activity in the control of mitochondrial function.

### Inhibition of MCAT protein by cAMP

**Figure 4:**
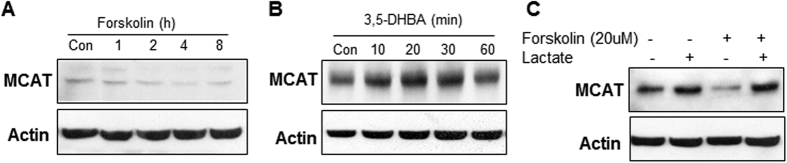
Inhibition of MCAT protein by cAMP pathway . (**A**) Reduction of MCAT protein by forskolin treatment (20 μM). MCAT protein was determined in C2C12 cells after forskolin treatment in Western blot. (**B**) Induction of MCAT protein by GPR81 activator. The test was performed in C2C12 cells after 3,5-DHBA treatment (1 mM). (**C**) Inhibition of forskolin activity by lactate. MCAT protein was determined in C2C12 cells after lactate pretreatment for 2 h followed by 4 h forskolin treatment.

MCAT is important in the maintenance of mitochondrial function, but the signaling pathway remains unknown in the regulation of MCAT activity. To address this issue, the cAMP-PKA pathway was investigated in the regulation of MCAT using the forskolin model (Fig. 4A). MCAT protein was reduced by forskolin activation of the cAMP pathway at 1 h, and the effect was maintained up to 8 h in the study. In contrast, the protein level was increased by down-regulation of the pathway from GPR81 activation by 3,5-DHBA (Fig. 4B). The increase was observed at 10 min with the treatment by GPR81 activator in the 60 min study (Fig. 4B). The peak increase was observed at 20 min. The MCAT inhibition by forskolin was significantly attenuated by pre-treatment of C2C12 cells with lactate (Fig. 4C). These data suggest that MCAT protein is reduced by the cAMP pathway, and is up-regulated upon inhibition of the pathway by lactate or 3,5-DHBA.

### Difference of pyruvate and lactate

**Figure 5:**
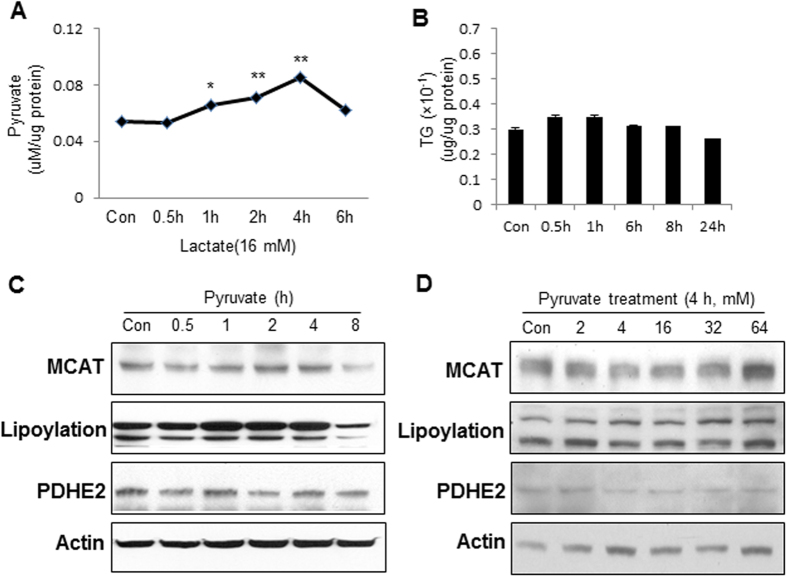
Pyruvate activity in C2C12 cells. (**A**) Induction of intracellular pyruvate by lactate. Pyruvate was examined in C2C12 cells treated with 16 mM lactate for different times. (**B**) TG in whole cell lysate. Total triglyceride was determined in C2C12 cells after pyruvate treatment (16 mM) at different times. (**C**) Time course of MCAT activity. MCAT protein and lipoylation signals were determined in pyruvate-treated cells by Western blot. (**D**) Does-dependent study of pyruvate. MCAT protein and lipoylation signals were determined after pyruvate treatment at different concentrations (2–64 mM) for 4 h. The data are presented as mean ± SD (n = 3) in the bar figure *p <0.05, **p <0.01.

The GPR81 receptor represents a receptor-dependent pathway for lactate in the regulation of TG accumulation and mitochondrial function. The receptor-independent pathway was investigated with a focus on pyruvate. Intracellular pyruvate was increased in C2C12 cells by the lactate treatment (Fig. 5A). The increase may mediate the lactate activity as well. To test this possibility, we treated C2C12 cells with pyruvate at a concentration (16 mM) with different duration (0.5–8 h), which mimic in the condition in the study of lactate activities. TG and MCAT were examined to determine the pyruvate effects. The TG level was not increased by pyruvate in the time course study (Fig. 5B). The protein level of MCAT and lipoylation signals (PDH and αKDH) were not significantly induced by pyruvate (Fig. 5C). The lipoylation signals were even reduced by pyruvate at 8 h in the study. No effect was observed for pyruvate in the regulation of MCAT protein and the lipoylation signals in C2C12 cells in the dose-dependent study (Fig. 5D). A mild reduction was observed in MCAT protein upon pyruvate treatment at 4 mM concentration. These data suggest that pyruvate may not mediate the lactate activity in the induction of TG accumulation and MCAT protein.

### Induction of GPR81 by lactate

**Figure 6:**
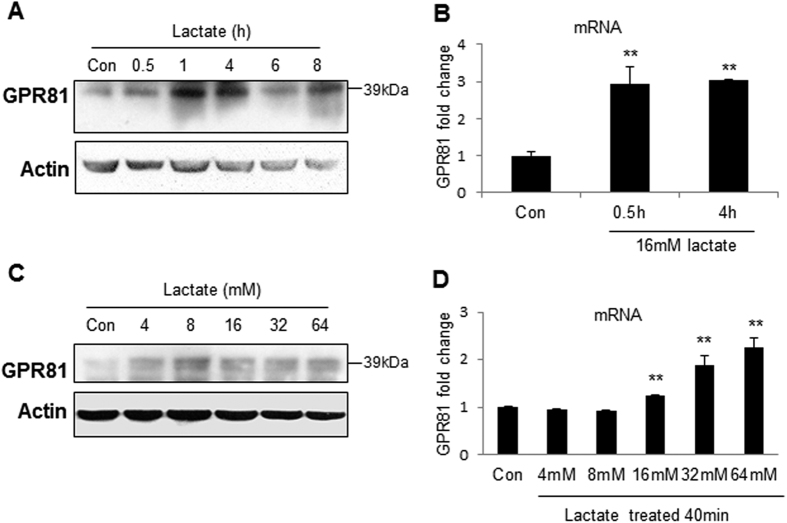
Induction of GPR81 expression by lactate in C2C12 cells. (**A**) Lactate induction of GPR81 protein. GPR81 protein was determined in the whole cell lysate of C2C12 by Western blot after lactate treatment (16 mM) at different times. (**B**) mRNA of GPR81. mRNA was quantified in cells at 0.5 h and 4 h after lactate treatment. (**C**) GPR81 protein in a dose-dependent study of lactate. The protein was determined in C2C12 cells after lactate treatment at concentrations of 4–64 mM for 2 h. (**D**) mRNA in a dose-dependent study of lactate. The cells were treated with lactate for 40 min at different concentrations (4–64 mM). The data are expressed as mean ± SD (n = 3). Statistical analysis was performed with Student’s test. *p < 0.05 and **p < 0.01 versus control group.

The factors that regulate GPR81 receptor expression remain largely unknown. To address this issue, protein and mRNA of GPR81 were examined in C2C12 cells following the lactate treatment. In the time course studies, the cells were treated with 16 mM lactate for different times. GPR81 protein was induced at 1 h of lactate treatment (Fig. 6A). The increase was associated with elevated expression of GPR81 mRNA. The mRNA was induced by lactate as early as 0.5 h in the treatment, and the lactate activity was observed at all of time points in the 4 h study (Fig. 6B). In the dose-dependent study, the lactate activity was observed at 4 mM with the highest activity at 8 mM (Fig. 6C). GPR81 mRNA was induced by lactate at concentrations of 16–64 mM in the 40 min study (Fig. 6D). No effect was observed for lactate at concentrations of 4–8 mM. These data suggest that lactate induces GPR81 expression in C2C12 cells.

### Induction of MCT1 and MCT4 expression by lactate

**Figure 7:**
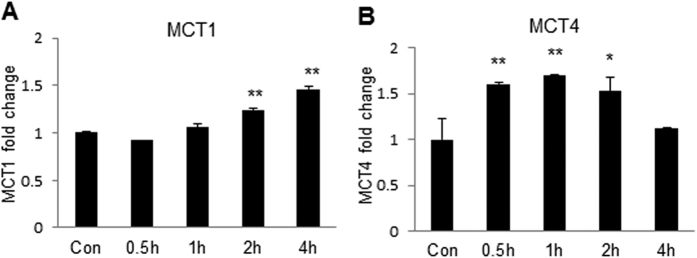
Induction of transports MCT1 and MCT4 by lactate. mRNA of MCT1 and MCT4 was determined in C2C12 cells after lactate treatment. (**A**) Time course study of MCT1 mRNA. The level was determined by qRT-PCR at time points as indicated after 16 mM lactate treatment. (**B**) Time course study of MCT4 mRNA. Relative mRNA level of MCT4 was determined after 16 mM lactate treatment. The data are expressed as mean ± SD (n = 3). Statistical comparisons are performed with Student’s test. *p < 0.05 and **p < 0.01 versus control group.

MCT1 and MCT4 are proton-coupled transporters of lactate, which are required for cross-membrane transport of lactate in cells. MCT1 involves in lactate uptakes by cells, and MCT4 is for export of lactate from cells^19^. To determine the role of lactate in the regulation of the transporters, we examined mRNA of MCT1 and MCT4 in C2C12 cells following the lactate treatment. MCT1 expression was induced by lactate (Fig. 7A) and the peak induction was observed at 4 h. MCT4 expression was induced as well, but the peak was observed at 0.5–1 h (Fig. 7B). These data suggest that lactate induces expression of MCT1 and MCT4, and the dynamics are different in the expression of two transporters upon lactate stimulation.

## Discussion

Our data suggests that lactate increases TG accumulation and mitochondrial function in myotubes in cell culture, which was observed at the physiological concentrations of lactate. TG accumulation is increased and mitochondrial capacity is enhanced in muscle by exercise^20^,^21^. However, the mechanism of the two events remains unknown. In this study, we tested lactate in the regulation of both events in mouse C2C12 myotubes, a cellular model. The data suggest that lactate has a signaling activity in the regulation of two events.

Our data suggest that lactate may activate the receptor GPR81 to induce the two events. Lactate activates GPR81 in adipocytes to induce TG accumulation by suppression of the cAMP-PKA pathway^7^,^8^,^9^,^22^. In this study, the pathway was tested in muscle cells. The data suggest that the same signaling pathway acts in muscle cells. In this study, expression of GPR81 was observed in C2C12 cells, and the cAMP-PKA pathway was inhibited at the downstream of GPR81. Lactate acted on GPR81 to inhibit the cAMP pathway as suggested by the activity of positive ligand of GPR81 (3,5-dihydroxybenzoic acid). Lactate blocked the forskolin activity in the activation of cAMP-PKA pathway. These data suggest that the GRP81 receptor may mediate the lactate activity in the down-regulation of the cAMP-PKA pathway in muscle cells. The pathway provides a mechanism for induction of TG accumulation by lactate in C2C12 myotubes. GPR81 was reported to induce ERK phosphorylation in adipocytes in some studies^7^,^17^. However, this effect was not strong in C2C12 cells, which may reflect a difference between myotubes and adipocytes.

Our data provide a mechanism for regulation of mitochondrial function by lactate. The lactate activity was investigated with a focus on MCAT, which is required for the maintenance of mitochondrial function^13^,^18^,^23^. Although the MCAT activity is known in mitochondria, it is not known how MCAT activity is regulated. Our data suggest that MCAT protein is induced by lactate through a mRNA-independent mechanism since MCAT mRNA was not up-regulated by lactate. In addition, our data suggests that the MCAT activity is inhibited by the cAMP pathway as suggested by the forskolin activity. Lactate may induce MCAT protein by blocking the cAMP activity. The change in MCAT activity was observed in protein, but not in mRNA expression. The enzyme activity of MCAT was induced by lactate as indicated by the alteration in lipoylation of PDH as well as αKDH, which are indicators of mitochondrial function.

Induction of GPR81 by lactate provides a mechanism of a positive feedback of lactate pathway. GPR81 mediates lactate signal to inhibit the cAMP-PKA pathway, but regulation of GPR81 expression has not been documented in the literature. Kashan, *et al.* found that lactate activated GPR81 with EC_50_ value of 1.5 mM in both human and mouse using GTP_γ_S binding methods^9^. Changlu, *et al*. reported that lactate stimulated the internalization of GPR81 and GTP_γ_S binding with EC_50_ value of about 5 mM^7^. EC_50_ value of lactate in the activation of GPR81 is 1.5–3 mM in adipocytes. Our results suggest that GPR81 expression is induced in protein and mRNA by lactate at 4–64 mM. The induction was consistent in the time course and dose-dependent studies of lactate. The GPR81 responses suggest a positive feedback regulation of the pathway by lactate in muscle cells.

Our data suggests that the lactate effect is not dependent on pyruvate. Lactate is converted into pyruvate for aerobic oxidation via the citric acid cycle in the presence of oxygen^4^,^6^. The effect of pyruvic acid was tested in this study. Pyruvate didn’t exhibit the same activity as lactate in the induction of TG accumulation and MCAT activity. Pyruvate enters mitochondria through two mitochondrial pyruvate carriers (MPCs)^24^,^25^, which serves as pyruvate transporters in mitochondria. In mitochondria, pyruvate is oxidized by the pyruvate dehydrogenase complex (PDH) to form acetyl-CoA^26^. Our data suggest that pyruvate may not involve in the signaling activity of lactate in muscle cells.

Lactate induced the mRNA expression of lactate transporters, MCT1 and MCT4. Proton-linked monocarboxylate transporters (MCTs)^27^ transport lactate and other intermediates metabolites such as pyruvate and ketone bodies across the cell membranes in mouse^28^,^29^ and human cells^30^. MCT1 mainly involves in cellular import of lactate in skeletal muscle^31^. While, MCT4 is highly expressed in glycolytic tissues (e.g. white muscle) for cellular export of lactate^32^. Regulation of MCT expression by a variety of stimuli has been reported, especially for MCT1 and MCT4 in skeletal muscle^33^. Mohammed S., *et al*. found that MCT4 expression was induced by lactate in hypoxia conditions to export lactate from the cells^34^. Audrey, *et al*. found that lactate was transported into cells through MCT1 in white adipocytes to stimulate Ucp1 expression^35^. In our study, lactate induced the mRNA level of both MCT1 and MCT4 although the dynamics were different. The data suggest that lactate may enhance shuttling of lactate and other metabolites (pyruvate and ketone bodies) in cells.

**Figure 8:**
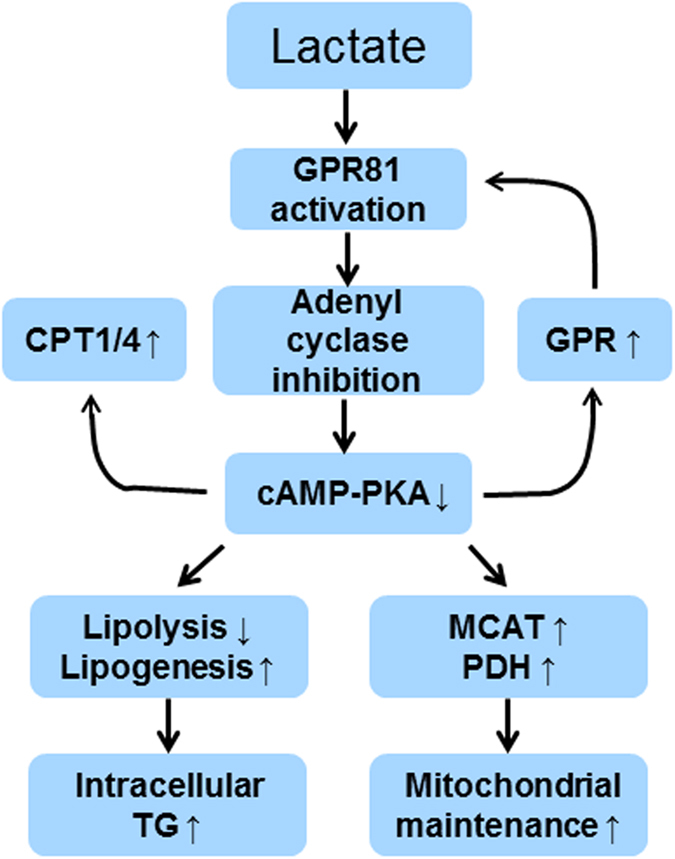
Signaling pathway and action of lactate in myotubes.

In conclusion, the current study suggests that lactate may sever as a signaling molecule in muscle cells to induce intramuscular triglyceride storage and maintenance of mitochondrial function. The study provides an outline of the signaling pathway of lactate, in which lactate activates the cell surface receptor GPR81 leading to inhibition of the cAMP pathway (Fig. 8). Inhibition of the cAMP pathway represents a mechanism of the lactate activities in the up-regulation TG accumulation and maintenance of mitochondrial function. The cAMP inhibition also provides a mechanism of the lactate activities in the up-regulation of GPR81, MCAT, MCT1 and MCT4. The GPR81 response suggests a positive feedback regulation of the lactate activities. These activities of lactate are likely independent of pyruvate.

### Research design and methods

#### Cell Culture

The mouse myoblast cell line C2C12 (CRL-1772), human embryonic kidney (HEK) 293 cells (CRL-1573) and mouse fibroblast cell line 3T3-L1 (CL-173) were purchased from the American Type Culture Collection (Manassas, VA). Cells were maintained in a high glucose DMEM (GIBCO 12800-017) supplemented with 10% fetal bovine serum (FBS) at 37 °C in a 5% CO_2_ incubator, and routinely examined for mycoplasma contamination. At 90-95% confluence in the 6 wells plate, C2C12 cells were differentiated into myotubes with 2% horse serum in DMEM. 3T3-L1 cells were differentiated into adipocytes using the adipogenic cocktail (10 μg/ml insulin, 0.5 mM isobutyl methylxanthine, and 10 μM dexamethasone) in 10% FBS DMEM after confluence. The cells were treated with lactate or pyruvate in serum-free and pyruvate-free DMEM (GIBCO 11960) containing 0.25% fatty acid–free bovine serum albumin (BSA).

#### Cell treatments

After differentiation, myotubes were serum-starved overnight in pyruvate-free medium, and then treated with sodium lactate (L7022, Sigma), forskolin (F6886, Sigma) or sodium pyruvate (P2256, Sigma-Aldrich). Sodium lactate and sodium pyruvate were prepared in H_2_O at a stock concentration of 1 M. Forskolin was prepared in DMSO at a stock concentration of 100 mM. The GPR81 ligand 3,5-dihydroxybenzoic acid (3,5-DHBA, D110000 Aldrich) was prepared in H_2_O at a stock concentration of 1 M. 293 cells and adipocytes were serum-starved overnight in 0.25% BSA medium before the treatments.

#### Oil Red O Staining

Intracellular lipid droplets were detected with oil-red O staining using a protocol as previously reported^36^. Myotubes were treated with sodium-lactate (16 mM and 20 mM) for 4 h. After incubation, cells were fixed with 10% paraformaldehyde for 30 min, and stained with a freshly-prepared working solution of oil-red O (3 mg/ml) for 15 min at the room temperature. After several times of washes, stained cells were observed under a microscope, and images were taken using a CellSens standard system (OLYPUS IX51, Tokyo, Japan).

#### Triglyceride assay

The total triglyceride and glycerol were determined in myotubes using the triglyceride determination kit (TR0100, Sigma) according to the manufacturer’s instruction. Briefly, the cells were harvested and sonicated in the whole lysate buffer. In the TG assay, samples was mixed with 40 μl of triglyceride reagent in the well, incubated for 15 min at 37 °C, and then measured for the light absorbance. In the glycerol assay, samples (10 μl) were added into the 160 μl of free glycerol reagent, incubated for 15 min at 37 °C, then subjected to the absorbance (OD540 nm) measurement using a microplate reader (Benchmark Plus Microplate Spectrophotometer, BIO-RAD, California, USA). The relative triglyceride and glycerol concentrations were normalized by the protein concentration. For all measurements, at least four biological replicates were used.

#### Protein extracts

Cells were harvested and suspended in the whole cell lysis buffer (50 mM KCl, 1% NP-40, 25 mM HEPES pH 7.8, 10 μg/ml leupeptin, 20 μg/ml aprotinin, 125 μM DTT, 1 mM PMSF, 1 mM Na_3_VO_4_). The whole cell lysate was sonicated in the lysis buffer, and centrifuged at 12000 g for 15 mins at 4 °C. The supernatant was collected and saved as the whole cell extracts. The concentration of protein was measured using the Pierce^TM^ BCA Protein Assay Kit (Prod# 23225, Life technologies, Eugene, Oregon) with BSA as a protein standard.

#### Western blot analysis

The cell protein extracts (50–100 μg/lane) were used in Western blotting according to a protocol described elsewhere^37^. Briefly, protein samples were separated in an 8% SDS-PAGE gel and transferred to the nitrocellulose membrane. After blocking in 5% nonfat milk buffer containing 0.05% Tween-20, the membrane was incubated with the primary antibody. The primary antibody to lipoylation (437695) was purchased from Calbiochem^®^ (Darmstadt, Germany); the antibody to phospho-p44/42 MAPK (pErk1/2, Thr202/Tyr204) was purchased from the Cell Signaling Technology (#9106, Danvers, MA, USA); the antibody to pDHE2 (ab66511) and CREB (ab7540) were obtained from Abcam (Cambridge, MA, USA); the antibody to pCREB-1 (Ser 133, sc-101663), GPR81 (T-16, sc-32647), β-actin (C4, sc-47778), MCAT (H-146, sc-366137) and ERK2 (D-2, sc-1647) were obtained from the Santa Cruz Biotechnology (Dallas, TX). β-actin were used as a protein loading control. The signal was visualized in a film after exposure to chemiluminescence and analyzed for optical densities at each band using an image-processing and analysis system.

#### Quantitative real-time RT-PCR

Total RNA was isolated from cells using the TRIzol reagent (Invitrogen, Carlsbad, CA) according to the manufacturer’s protocol. mRNA (100 ng/μl) was reverse transcribed using the High-Capacity cDNA Reverse Transcription Kit (4368814, Life Technologies/Applied Biosystems). TaqMan Universal PCR Master Mix (4304437; Applied Biosystems, Carlsbad, CA) was used to quantify mRNA using the ABI 7900 machine. MCAT mRNA was examined using the TaqMan protocol and normalized to ribosomal 18S1 RNA (Mm04277571-s1). The primer of MCAT (Mm01299438_m1) was purchased from the Applied Biosystems. mRNA of GPR81, MCT1 and MCT4 was determined using the SYBR green qRT-PCR protocol. The primers were ordered from Sigma-Aldrich and master mix reagent was from Applied Biosystems (4309155, Applied Biosystems). The mRNA signal was normalized to GADPH. The primer sequences are listed in table 1 below. The relative gene expression of target mRNA was calculated by ΔΔCT method.

#### PDH activity assay

The catalytic activity of PDH was determined in myoblasts using the Pyruvate Dehydrogenase (PDH) Enzyme Activity Microplate Assay Kit (ab109902, Abcam) according to the manufacturer’s instruction. Briefly, the cell lysate was diluted to a final concentration of 2.5 μg/μl. The sample was added into each well in microplate at 200 μl and was incubated for 3 h at room temperature. The reaction then was stabilized and incubated with the assay buffer. The absorbance of each well was determined at 450 nm at room temperature for 60 min with 10s interval using a kinetic program. The slopes of the curves were generated to express the PDH activity.

#### Pyruvate assay

The pyruvate concentration in myotubes was determined using the Pyruvate Colorimetric/Fluorometric Assay Kit (K609-100, BioVision, Milpitas CA, USA) according to the manufacturer’s instruction. Briefly, the standard curve was made with pyruvate at 0, 2, 4, 6, 8, and 10 nM, and the correlation coefficient was 0.995 or higher. Pyruvate was extracted from cells with 4 volumes of the Pyruvate Assay Buffer. Supernatant was collected and added into the reaction mix at 10 μl/well in the colorimetric assay. The reading of absorbance (OD570 nm) was taken using a microplate reader (Benchmark Plus Microplate Spectrophotometer, BIO-RAD, California, USA). The relative pyruvate concentration was normalized by the sample’s protein concentration.

#### Statistical Analysis

Two-tailed, unpaired Student t test was used to calculate p value. p < 0.05 was considered significant, P < 0.01 was considered very significant. All the results were presented as mean ± SEM.

## Additional Information

**How to cite this article**: Sun, J. *et al.* Induction of triglyceride accumulation and mitochondrial maintenance in muscle cells by lactate. *Sci. Rep.* **6**, 33732; doi: 10.1038/srep33732 (2016).

## Figures and Tables

**Table 1:** List and sequences of primers.

Target name	Forward primer	Reverse primer
GPR81	5-GGCTGAGAAAAGCGGTATGA-3	5-TCGTTAACTCTCTCCGAGCTAGA-3
mMct1	5-GAGGTTCTCCAGTGCTGTG-3	5-TCCATACATGTCATTGAGGCG-3
mMct4	5-AGTGCCATTGGTCTCGTG-3	5-CATACTTGTAAACTTTGGTTGCATC-3
GADPH	5-GACGGCCGCATCTTCTTGT-3	5-CACACCGACCTTCACCATTTT-3
